# VEGFA165 gene therapy ameliorates blood-labyrinth barrier breakdown and hearing loss

**DOI:** 10.1172/jci.insight.143285

**Published:** 2021-04-22

**Authors:** Jinhui Zhang, Zhiqiang Hou, Xiaohan Wang, Han Jiang, Lingling Neng, Yunpei Zhang, Qing Yu, George Burwood, Junha Song, Manfred Auer, Anders Fridberger, Michael Hoa, Xiaorui Shi

**Affiliations:** 1 Oregon Hearing Research Center, Department of Otolaryngology-Head & Neck Surgery, Oregon Health & Science University, Portland, Oregon, USA.; 2 Boston Children’s Hospital, Harvard Medical School, Boston, Massachusetts, USA.; 3 Life Sciences Division, Lawrence Berkeley National Laboratory, Berkeley, California, USA.; 4 Department of Biomedical and Clinical Sciences, Linköping University, Linköping, Sweden.; 5 Auditory Development and Restoration Program, National Institute on Deafness and Other Communication Disorders (NIDCD), NIH, Bethesda, Maryland, USA.

**Keywords:** Angiogenesis, Cardiovascular disease

## Abstract

Millions of people are affected by hearing loss. Hearing loss is frequently caused by noise or aging and often associated with loss of pericytes. Pericytes populate the small vessels in the adult cochlea. However, their role in different types of hearing loss is largely unknown. Using an inducible and conditional pericyte depletion mouse model and noise-exposed mouse model, we show that loss of pericytes leads to marked changes in vascular structure, in turn leading to vascular degeneration and hearing loss. In vitro, using advanced tissue explants from pericyte fluorescence reporter models combined with exogenous donor pericytes, we show that pericytes, signaled by VEGF isoform A165 (VEGFA165), vigorously drive new vessel growth in both adult and neonatal mouse inner ear tissue. In vivo, the delivery of an adeno-associated virus serotype 1–mediated (AAV1–mediated) VEGFA165 viral vector to pericyte-depleted or noise-exposed animals prevented and regenerated lost pericytes, improved blood supply, and attenuated hearing loss. These studies provide the first clear-cut evidence that pericytes are critical for vascular regeneration, vascular stability, and hearing in adults. The restoration of vascular function in the damaged cochlea, including in noise-exposed animals, suggests that VEGFA165 gene therapy could be a new strategy for ameliorating vascular associated hearing disorders.

## Introduction

Adequate blood supply is fundamental for auditory function since the processes responsible for hearing in the inner ear demand large amounts of energy. Thus, maintenance of normal blood flow to the ear is critical for hearing. The most critical microvascular network in the cochlea is located in the cochlear stria vascularis. This vascular network receives the larger portion of cochlear blood flow (1). It is essential for cochlear fluid ion homeostasis, particularly for generating the endocochlear potential (EP) (2), on which mechanotransduction by hair cells (HCs) depends. The microvascular beds in this region also constitute a tightly controlled blood-labyrinth barrier, with a rich population of pericytes, providing a timely supply of oxygen and nutrients to the cochlea (3).

Numerous functions have been ascribed to pericytes in different organ systems, including vascular development, blood flow regulation, vascular integrity, and angiogenesis (4–7). The microvasculature in the stria vascularis of the ear contains a high density of pericytes (3), but the role of pericytes in cochlear homeostasis has received little attention. Here, using a conditional pericyte depletion adult animal model, we report that pericytes are essential for the stability of mature vessel beds and vascular function. Loss of pericytes results in marked damage to vascular architecture and blood-barrier integrity, reduced blood supply to the cochlea, and sensory HC death. The culmination of these changes is loss of hearing sensitivity. Urgent questions need to be asked: Can lost pericytes be restored? Does restoration of lost pericytes improve damaged vascular function and help recover lost hearing sensitivity? A recent study from our lab demonstrates that VEGF isoform A165 (VEGFA165) induces cochlear angiogenesis in tissue culture plates (8). In another study, VEGFA was shown to exert direct effects on pericytes to promote pericyte proliferation (9). Can VEGFA, by affecting the cellular status of pericytes, boost the regenerative capacity that drives new vessel growth in the mature cochlear vascular system, particularly under hypoxic conditions? Contrary to the prevailing view that specialized tip cells derived from endothelial cells guide angiogenesis (10–12), we demonstrate in this study that VEGFA165 stimulates cochlear pericytes as the leading tip cells to guide new vascular branch growth in both adult and neonate mouse cochleae. Pharmacological depletion of pericytes dramatically halts the growth of new vessel sprouts, while the addition of exogenous pericytes to the tissue markedly promotes the growth of new sprouts, demonstrating that a normal population of pericytes is essential for vascular dynamics. We then further tested whether VEGFA165 promotes pericyte regeneration in vivo and found that the delivery of adeno-associated virus serotype 1–VEGFA165 (AAV1–VEGFA165) to pericyte-depleted adult animals promotes the regeneration of pericytes. As a consequence of the treatment, vascular damage is attenuated and lost blood supply to the cochlea is ameliorated. With the restoration of lost pericytes and blood flow, loss of sensory HCs is mitigated and hearing sensitivity is significantly improved.

This is the first study to our knowledge to demonstrate the critical role of pericytes and VEGFA signaling in support of vascular function and hearing in the adult inner ear. We show that pericyte depletion and regeneration have significant effects on vascular function and hearing outcome. Since disruption of cochlear blood flow is found in many forms of hearing loss, including in presbycusis and noise-induced and viral infection–induced hearing loss (13–16), the effectiveness of AAV1–mediated VEGFA165 gene therapy suggests a new treatment strategy. The effectiveness against noise-induced hearing loss, such as we demonstrated in this study, may generalize to other types of hearing loss. It may also be relevant to developing improved therapies for preventing neuronal loss in the central nervous system, as pericyte loss causes circulatory failure and is associated with multiple neurological disorders (17).

## Results

### Loss of pericytes impairs vascular structure and causes strial blood-barrier breakdown.

**Figure 1:**
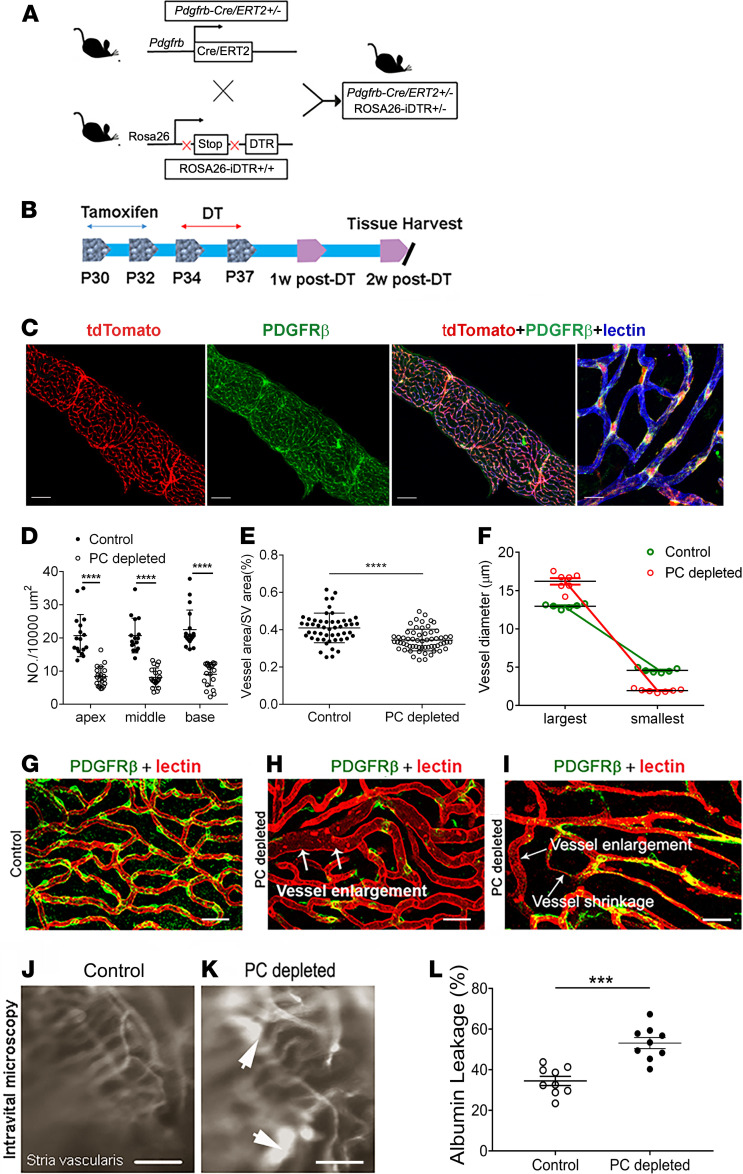
Loss of pericytes impairs vascular structure and function. (**A**) Schematic of the transgenic *Pdgfrb-CreERT2*/iDTR mouse model incorporating an inducible Cre-*loxP* system. (**B**) The diagram shows the timeline of tamoxifen and diphtheria toxin (DT) administration and the time point for tissue harvest. (**C**) Colocalization of Cre and PDGFR-β signals (green) is seen in the strial vasculature of *Pdgfrb-CreERT2*/tdTomato mice (middle right and right: low-magnification and high-magnification images, *n* = 4). (**D**) Pericyte density in the strial vasculature is significantly reduced in pericyte-depleted mice (*n* = 7) compared with control mice (*n* = 6) at the apical, middle, and basal turn (****P < 0.0001 by Student’s *t* test). (**E**) Total vascular density in the stria is also significantly reduced in pericyte-depleted mice (****P < 0.0001 by Student’s *t* test). (**F**) Maximum and minimum vessel diameter in control and pericyte-depleted animals (maximum diameter is approximately 12.9 ± 1.8 μm in control mice (*n* = 6, total 153 vessels analyzed), 16.2 ± 2.6 μm in pericyte-depleted mice (*n* = 7, total 180 vessels analyzed); minimum vessel diameter is approximately 4.6 ± 1.1 μm in control mice, 1.9 ± 0.8 μm in pericyte-depleted mice. (**G**–**I**) Representative figures show the capillaries of the stria in control (**G**) and pericyte-depleted mice 2 weeks after DT injection (arrows, **H** and **I**). The depletion of pericytes leads to some vessel enlargement (arrows, **H**) and shrinkage (arrows, **I**). (**J** and **K**) Albumin-FITC leakage was identified under IVM in pericyte-depleted mice (white arrows, **K**) but not in the control animals (**J**). (**L**) Statistical difference in vascular leakage between control and pericyte-depleted animals (*n* = 3, ***P < 0.001 by Student’s *t* test). Data are presented as mean ± SEM. Scale bars: 10 μm (**C** left, middle left, middle right), 30 μm (**C** right). 20 μm (**G**, **H**, **I**). 50 μm (**J**, **K**).

In this study, an inducible pericyte depletion mouse model (*Pdgfrb-CreERT2*/iDTR) was created by crossing *Pdgfrb-CreERT2* transgenic mice with inducible diphtheria toxin receptor (iDTR) mice (as illustrated in Figure 1A). This *Pdgfrb-CreERT2*/iDTR mouse line at 1 month of age received tamoxifen for 3 days to induce the expression of Cre recombinase (Figure 1B). The cellular location of Cre recombinase expression under the *Pdgfrb*-promotor was confirmed by crossing the *Pdgfrb-CreERT2* mice with RCL-tdTomato mice. As expected, the tdTomato fluorescence (4) signal exclusively colocalized with the immunofluorescence signal for the pericyte marker protein, PDGFR-β (shown in green), as shown in Figure 1C. Control iDTR and *Pdgfrb-CreERT2*/iDTR mice at P34 received intraperitoneal injections of DT at 10 ng/g body weight for 4 consecutive days (Figure 1B). In the *Pdgfrb-CreERT2*/iDTR mice, the pericyte population in the strial vascular networks was reduced at all cochlear turns 2 weeks after DT treatment (Figure 1D). Total vascular density in the stria was also significantly reduced in pericyte-depleted mice 2 weeks after DT treatment (Figure 1E). Vascular diameter was highly variable (Figure 1F). Compared with the highly organized vascular architecture in the control of iDTR mice (Figure 1G), the pericyte-depleted vasculature showed marked disruption. The enlargement of vessels where pericytes were lost was frequently observed (Figure 1H). Occasionally, we also observed vascular shrinkage, a sign of degeneration, when pericytes were abnormal or absent (Figure 1I). Concomitant with the vascular damage, examination with high-resolution intravital fluorescence microscopy (IVM) showed marked vascular leakage in the pericyte-depleted animals. Figure 1, J and K, show vascular leakage in control and pericyte-depleted animals, respectively. Vascular leakage of albumin was significant (Figure 1L). Collectively, our results strongly indicate that pericytes are essential for vascular stability and functional integrity in mature animals. The loss of pericytes precedes vascular degeneration and leads to blood-barrier breakdown.

### Loss of pericytes causes hearing loss.

**Figure 2:**
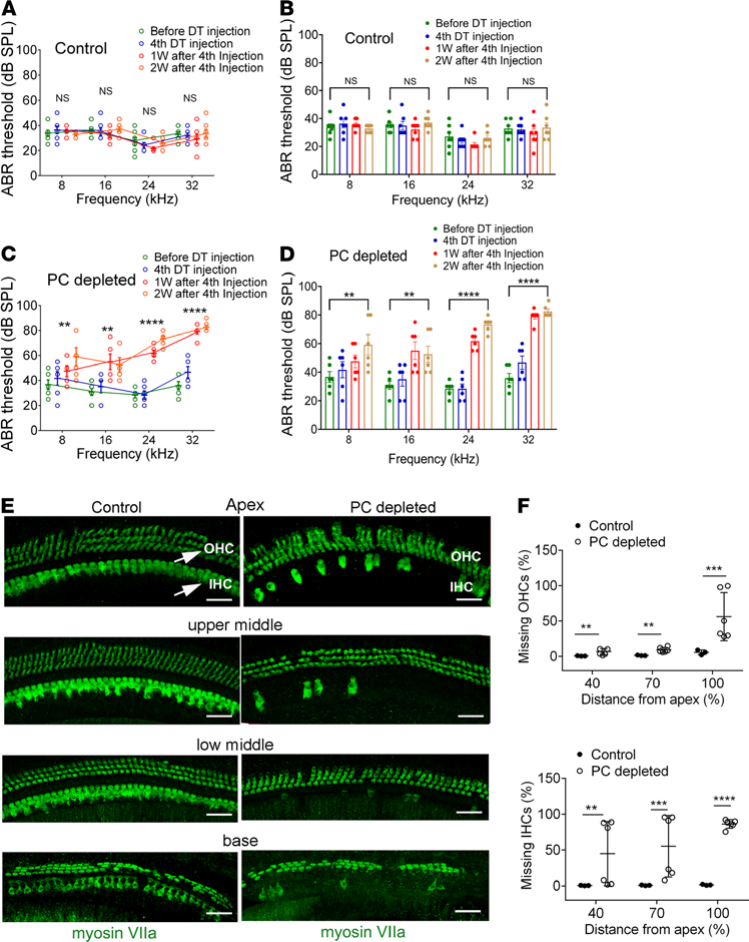
Loss of pericytes causes hearing loss. (**A** and **B**) Control iDTR mice receiving the same 4 doses of DT as the *Pdgfrb-CreERT2*/iDTR mice showed no significant hearing threshold change at 1 week and 2 weeks (*n* = 7, *P* > 0.05 by repeated measures 1-way ANOVA). (**C** and **D**) The depletion of pericytes led to hearing loss at all measured frequencies. The hearing threshold in pericyte-depleted animals was significantly elevated at 1 week after DT injection (*n* = 6, **P < 0.01, and ****P < 0.0001) and persisted 2 weeks after DT injection (*n* = 6, **P < 0.01, and ****P < 0.0001 by repeated measures 1-way ANOVA). (**E** and **F**) Significant HC loss is seen at the middle and basal turns in the pericyte-depleted animals. (**E**) Representative high-magnification confocal images from control and pericyte-depleted animals, labeled with antibody for myosin VIIa in the different cochlear regions. (**F**) Total percentage of inner HC (IHC) and outer HC (OHC) (see arrows in **E**) loss along the entire length of the cochlea in each group was calculated. The pericyte-depleted group showed significantly more IHC and OHC loss (*n_control_* = 3, *n*_PC_ _depleted_ = 6, **P *<* 0.01 for apical IHC loss, ***P = 0.001 for middle to base IHC loss, ***P < 0.001 for base to hook IHC loss, **P < 0.01 for apical OHC loss, **P < 0.01 for middle to base OHC loss, and ***P = 0.001 for base to hook OHC loss by unpaired Student’s *t* test). Scale bars: 30 μm.

The administration of DT to control mice showed no significant hearing threshold shift (Figure 2, A and B). In contrast, pericyte-depleted animals displayed a significant elevation of hearing threshold at all measured frequencies starting at 1 week after DT injection, with a higher elevation of the hearing threshold at 2 weeks (Figure 2, C and D). Pericyte-depleted animals also showed significant loss of sensory HCs, including both inner and outer HCs, particularly inner HCs (Figure 2E). Figure 2F, a representative confocal image from pericyte-depleted animals labeled with an antibody for myosin VIIa, shows HC loss at the different cochlear turns. In contrast, the distribution of HCs in control animals remained intact.

### Pericytes signaled by VEGFA165 have a strong vascular regenerative capacity in vitro.

**Figure 3:**
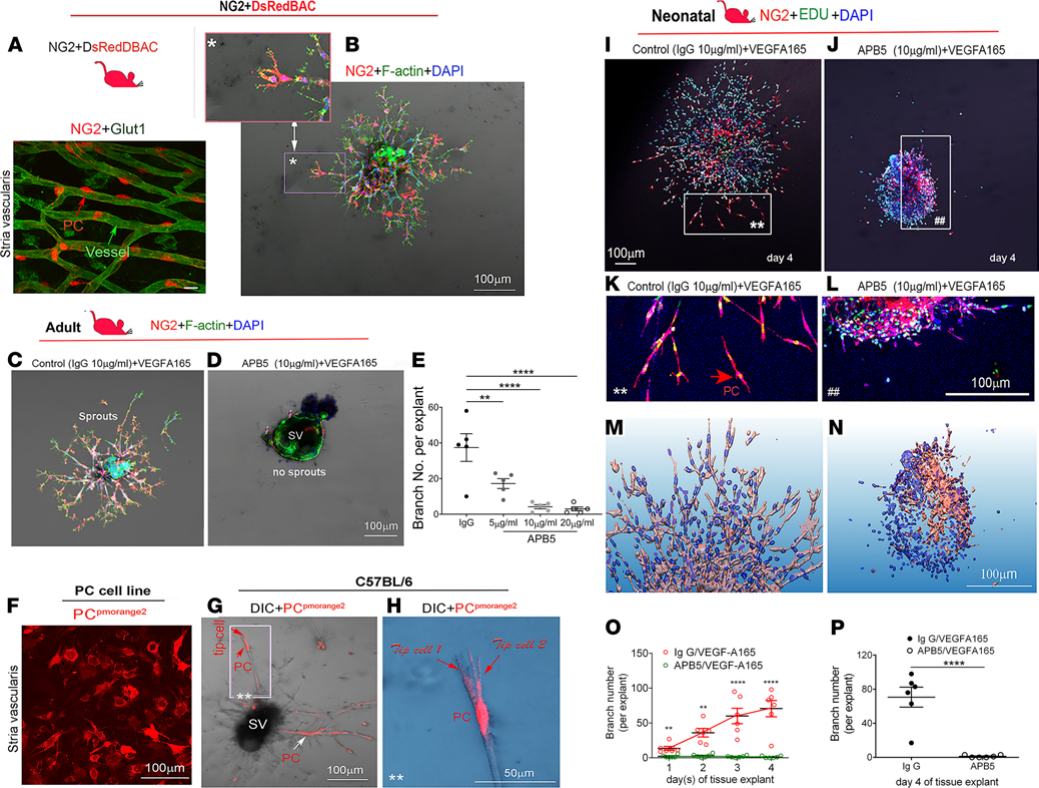
Pericytes, signaled by VEGFA165, lead cochlear angiogenesis in adult and neonatal mouse cochleae in vitro. (**A**) Confocal image of the stria from an NG2+DsRed mouse (*n* = 4) shows pericytes situated on microvessels labeled with an antibody for glucose transporter I (Glut, green). (**B**) Strial explant from an NG2+DsRed mouse cochlea labeled with Phalloidin and DAPI on day 5 after VEGFA165 treatment. A high-magnification image (inset in **B**) highlights how NG2-derived pericytes lead new vessel growth (*n* = 4). (**C**) An example of sprouting angiogenesis on day 5 from the control group. (**D**) Pericyte depletion with APB5 treatment impairs angiogenesis. (**E**) Quantitation of branch number in control and APB5-treated groups shows significant differences (*n* = 5 for each group, **P < 0.01, and ****P < 0.001 by 1-way ANOVA). (**F**) Pericytes encoded with pmOrange2-N1. (**G**) An explant cografted with fluorescence-labeled exogenous pericytes shows some pericytes convert to tip cells (red arrows) and some invest on sprouting branches (white arrows) on day 5 after treatment with VEGFA165. (**H**) A high-magnification image from an inset in **G** shows 2 exogenous pericytes with long filopodia situated on the distal end of sprouts. (Background color of inset in **H** was changed using Photoshop for better visualization of tip cells originating from donor pericytes.) (**I** and **J**) Confocal images show sprouting angiogenesis on day 4 in control and pericyte-depleted groups from neonatal mice. Zoomed-in images from insets in **I** and **J** better show NG2-derived pericyte-led branch formation in the control (**I**) and pericyte-depleted groups (**J**). (**M** and **N**) Reconstructions in 3D of confocal images from control and pericyte-depleted groups show less branch formation when pericytes are depleted. (**O**) Quantitative analysis of branch number in the 2 groups for each day (*n* = 6 for each group, **P < 0.01, and ****P < 0.0001 by Student’s *t* test) or (**P**) on day 4 (*n* = 6, ****P < 0.0001 by Student’s *t* test). Scale bars: 10 μm (**A**, **B** inset), 100 μm (**B**–**D**, **F**, **G**, **I**–**N**), 50 μm (**H**).

Pericytes are considered pluripotent progenitor cells that play an important role in vascular development and maintenance of vascular homeostasis (18). As shown above, pericyte depletion causes vascular damage and degenerative changes in the cochlea. Given the known role of pericytes in vascular branching morphogenesis (19), we asked whether pericyte control of vascular stability involved angiogenesis and whether it was signaled by VEGFA165. To answer the question, we used an NG2^+^ transgenic pericyte fluorescence reporter mouse expressing an optimized red fluorescent protein variant (DsRed.T1) under the control of an NG2 (*Cspg4*) promoter. In this mouse model, only pericytes showed NG2 fluorescence (DsRed.T1) in the adult cochleae (Figure 3A). Interestingly, by culturing strial tissue obtained from these mice (at ages ~4–8 weeks) in medium containing VEGFA165, we found the NG2^+^ pericytes, as leading cells, vigorously drove the sprouting angiogenesis. Figure 3B is a representative image showing the distal end of sprouting in an NG2^+^ pericyte. This phenomenon of pericytes leading angiogenesis is also seen in other mouse lines, including in the pericytes of a green fluorescence reporter NG2-ZsGreen (Supplemental Figure 1; supplemental material available online with this article; https://doi.org/10.1172/jci.insight.143285DS1) transgenic mouse line and a double fluorescence (green and red) reporter NG2^+^DsRedBAC/ZsGreen transgenic mouse line (Supplemental Figure 2). In the second set of experiments, we pharmacologically depleted pericytes with a neutralizing anti–PDGFR-β antibody, APB5, which specifically binds to PDGFR-β. The blockage of PDGF/PDGFR-β was previously reported to specifically ablate pericytes in the retina (20). In our study, we found that strial explants of the pericyte-ablated group showed a marked reduction in branch formation compared with the control group (Figure 3C). Some of the explants in the pericyte-ablated group showed no vessel pruning on day 5 in culture, as shown in Figure 3D. The branch number in the pericyte-ablated group was reduced in a dose-dependent manner (Figure 3E). Last, to confirm the effect, we performed a cell-tissue coculture experiment. Purified cochlear pericytes were tagged with the fluorescent protein pmOrange2-N1 (Figure 3F) and seeded around a strial explant on the fourth day in culture. We found the new vascular branches grafted by donor pericytes appeared stronger and longer, indicating exogenous pericytes promote new sprouting growth. Figure 3, G and H, show exogenous donor pericytes at the distal sprouting end with polarized filopodia. Other donor pericytes invested on vascular branches. Collectively, these data uncover some of the critical roles of pericytes in sprouting angiogenesis in the cochlear stria.

We also examined whether pericytes control angiogenesis in early vascular development. Blood vessels in the cochlear lateral wall are known to undergo angiogenesis from birth until P15 to form a highly organized vascular network (21). This time window provides us an ideal opportunity to capture the natural process of vascular formation. Strial tissue was isolated from P1 NG2^+^ transgenic mice and divided into 2 groups: VEGFA165 + control (IgG) and VEGFA165 + APB5. Consistent with our results obtained from adult mouse cochleae, we found NG2^+^ pericytes led angiogenesis in the neonatal mice. Figure 3, I and J, are representative confocal projection images showing sprouting angiogenesis in the control and APB5 groups on day 4 under low and high magnification (Figure 3, K and L). Figure 3, M and N, further show the spatial orientation and volumetric patterns of new branches in control and pericyte-depleted groups on day 4 using in-depth 3D reconstructions. Significant new branch growth was noticed in controls on day 4 compared with the APB5 group (Figure 3P). Figure 3O is a statistical comparison of the time course (days 1–4) of sprouting angiogenesis in the control and pericyte-depleted groups. Increasing new branch formation was noticed in the controls over time but not in the pericyte-depleted groups (representative confocal images in Supplemental Figure 3). Taken together, our data strongly indicate pericytes led to new sprouting growth under VEGFA165 signaling.

### Pericyte-driven angiogenesis is halted by blockage of VEGFA165 and boosted by promotion of VEGFA165.

**Figure 4:**
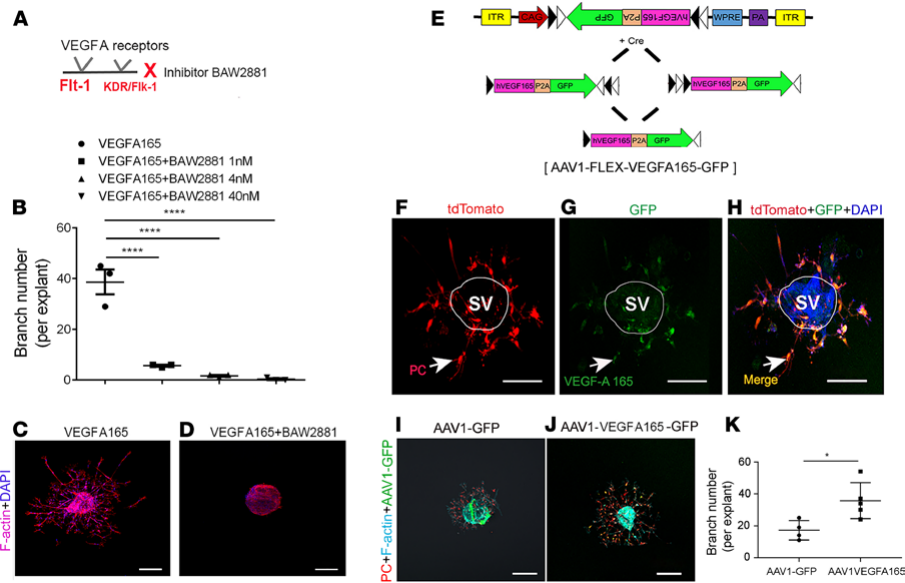
Pericyte-driven angiogenesis is controlled by VEGFA165 signaling. (**A**) The illustration shows selective blockage of VEGFA165 signaling with the drug BAW2881, which targets 2 VEGFA receptors, Flt1 and KDR/Flk1. (**B**) The number of new vessels is significantly reduced in a dose-dependent manner when VEGFA receptors are blocked with the specific VEGFA blocker, BAW2881 (*n* = 3 for control group, *n* = 4 for 1 nM, *n* = 3 for 4 nM, *n* = 3 for 40 nM, and ****P < 0.0001 by 1-way ANOVA). (**C** and **D**) Representative confocal projection images show branch formation in the VEGFA165 and VEGFA receptor blocked groups. (**E**) Illustration of construction of the AAV1-FLEX-VEGFA165-GFP viral vector, a Cre-dependent inversion gene switch (FLEX) vector, which specifically targets pericytes to produce VEGFA165. (**F**) Pericytes (red, genetically labeled by tdTomato) drive sprouting angiogenesis, as seen on day 5 in a *Pdgfrb-CreER*/tdTomato mouse cochlea. (**G**) The green AAV1-FLEX-VEGFA165-GFP signal is primarily targeted in tdTomato-labeled pericytes. (**H**) Merged image from **F** and **G**. (**I** and **J**) Sprouting growth in the AAV1-GFP control and AAV1-FLEX-VEGFA165 viral vector groups. (**K**) The number of branches is significantly higher in the AAV1-FLEX-VEGFA165 viral vector–transfected strial explant (*n* = 4 for AVV1-GFP group, *n* = 5 for AAV1-VEGFA165 group, and *P < 0.05 by Student’s *t* test). Data are presented as mean ± SEM. Scale bars: 100 μm (**C**, **D**, **F**–**H**), 150 μm (**I**, **J**).

To further investigate whether pericyte-induced angiogenesis is strongly dependent on VEGFA165 signaling, we used a VEGFA receptor blocker, BAW2881, to block the VEGFA receptors Flt1 and KDR/Flk1 (as illustrated in Figure 4A). We found the elimination of VEGFA signaling significantly suppressed new vascular branch formation in a dose-dependent manner (Figure 4B). Figure 4, C and D, are representative images showing active angiogenesis on day 5 in the VEGFA165-treated group but not in the VEGFA165 signal–blocked group. On the other hand, when we upregulated VEGFA165 using an AAV1-based Cre-dependent *Vegfa165* gene viral vector (gene vector designed using a FLEX strategy, as illustrated in Figure 4E) to specifically target pericytes in mouse cochleae, we found that VEGFA165 significantly promoted new branch growth formation. Figure 4, F–H, show pericytes (4) in strial tissue from a *Pdgfrb-CreER*/tdTomato mouse cochlea specifically infected with the AAV1-FLEX-*Vegfa165* viral vector. Figure 4, I and J, show the patterns of pericyte-driven sprouting angiogenesis in the AAV1-GFP (null gene) control and AAV1-FLEX-VEGFA165–treated groups. Figure 4K shows that the number of sprouts was significantly higher in the AAV1-FLEX-VEGFA165–transfected strial explant. Taken together, our data are strong evidence that VEGFA165 signaling is critical for pericyte migration and pericyte-driven angiogenesis.

### VEGFA165 enhances pericyte survival under hypoxic conditions in vitro.

**Figure 5:**
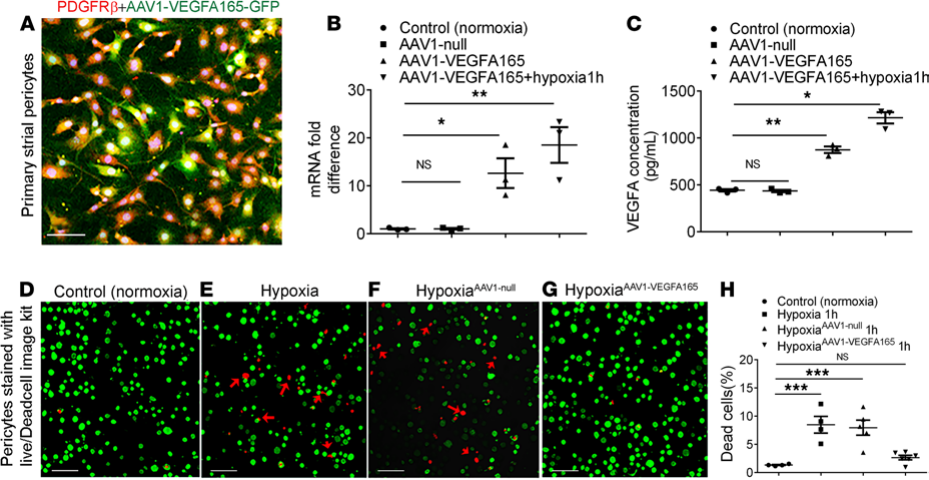
AAV1-HRE-VEGFA165 transfer to pericytes significantly promotes pericyte survival under hypoxic conditions. (**A**) AAV1-HRE-VEGFA165-GFP successfully infected a cochlear pericyte cell line with a multiplicity of infection (MOI) of 1 × 10^5^ when imaged 48 hours later. (**B**) Real time-quantitative PCR shows significant upregulation of *Vegfa* mRNA (*n* = 3 for each group, *P < 0.05, and **P < 0.01 by 1-way ANOVA). (**C**) ELISA shows high production of VEGFA protein in an AAV1-HRE-VEGFA165 gene-infected pericyte relative to an AAV1-GFP null-infected pericyte (*n* = 3 for each group, *P < 0.05, and **P < 0.01 by 1-way ANOVA). (**D**–**G**) Distribution of live (green) and dead pericytes under hypoxia as measured with a Live/Dead Cell Viability Assay Kit (MilliporeSigma). (**H**) Statistical analysis shows increased pericyte survival (green) under hypoxic conditions when pericytes were pretransfected with AAV1-HRE-VEGFA165 for 48 hours (*n* = 4 for control, *n* = 4 for hypoxia 1h, *n* = 5 for AAV1-GFP hypoxia 1h, *n* = 6 for AAV1-VEGFA165 hypoxia 1h, and ***P < 0.001 by 1-way ANOVA). Data are presented as mean ± SEM. Scale bars: 100 μm.

In addition to the promotion of pericyte-controlled angiogenesis, VEGFA165 upregulation was also found to protect pericytes and promote their survival under hypoxia. An AAV1-HRE-VEGFA165 viral vector, where the *Vegfa165* gene is under the control of a hypoxia response enhancer (HRE), was designed to upregulate the VEGFA165 expression in cells only under hypoxic conditions, thus avoiding overexpression of VEGFA165. Figure 5, A–C, respectively, show the AAV1-HRE-VEGFA165 vector successfully infected a primary pericyte cell line and caused high production of VEGFA165 mRNA and protein in the infected pericytes under hypoxic conditions relative to AAV1-null–infected pericytes. Figure 5, D–H, show AAV1-HRE-VEGFA165 transfection of pericytes significantly promoted pericyte survival.

### VEGFA165 promotes pericyte survival and proliferation and markedly attenuates vascular damage in vivo.

**Figure 6:**
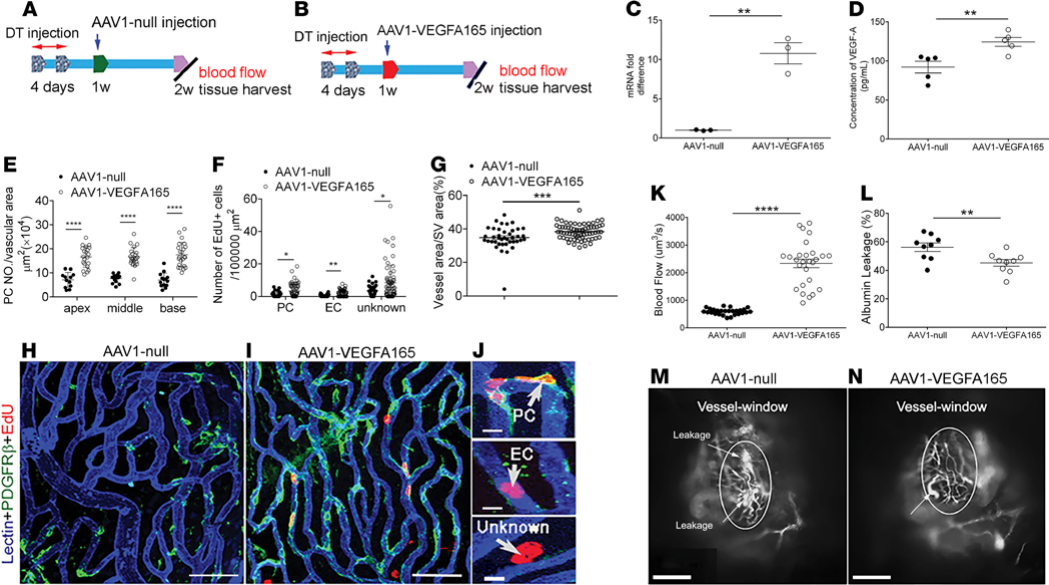
VEGFA165 gene therapy enhances pericyte survival, promotes pericyte regeneration, and attenuates vascular damage. (**A** and **B**) Time points of gene delivery and related measurements. (**C** and **D**) VEGFA165 mRNA and protein are significantly increased in the AAV1-VEGFA165 gene–treated cochleae compared with the control AAV1-treated cochleae (*VEGFA165* mRNA: *n* = 3 for each group; VEGFA165 protein: *n* = 6 for each group; and **P < 0.01 by Student’s *t* test). (**E**) The pericyte population is higher in the AAV1-VEGFA165 gene–treated group (*n* = 7) compared with the control AAV1-treated group (*n* = 4, ****P < 0.0001 by Student’s *t* test). (**F**) Increased EdU^+^ cells in the VEGFA165 gene–treated group (*n* = 7) compared with the control AAV1 group (*n* = 4, *P < 0.05, and **P < 0.01 by Student’s *t* test). (**G**) Decreased vascular density is attenuated by AAV1-VEGFA165 gene treatment (*n* = 7) but not by control AAV1 treatment (*n* = 4, ***P < 0.001 by Student’s *t* test). (**H** and **I**) Confocal images show EdU^+^ pericytes, endothelial cells, and an unidentified cell type in the stria of control AAV1 (**H**) and AAV1-VEGFA165 groups (**l**). (**J**) Zoomed-in images highlight different types of EdU^+^ cells in the stria of an AAV1-VEGFA165–treated animal. (**K**) Blood flow volume in the AAV1-VEGFA165–treated group is significantly greater than in the control AAV1 group (*n* = 3, ****P < 0.0001 by Student’s *t* test). (**L**) Vascular leakage is markedly attenuated in the AAV1-VEGFA165–treated group relative to the control AAV1 group (*n* = 3 for each group, **P < 0.01 by Student’s *t* test). (**M** and **N**) IVM images from control AAV1 and AAV1-VEGFA165 groups (white arrows, leaking sites). Data are presented as a mean ± SEM. Scale bars: 50 μm (**H**, **I**), 10 μm (**J**), 100 μm (**M**, **N**). PC, pericyte; EC, endothelial cell.

Can lost pericytes be restored? Does the restoration of lost pericytes improve damaged vascular function? An early study showed that VEGFA delivered at an appropriate microenvironmental concentration acts as a key regulator of vascular proliferation and new vessel formation (22). To test whether pericyte loss can be prevented or lost pericytes can be regenerated by VEGFA165, we specifically administered the AAV1-HRE-VEGFA165 or controlled AAV1 at the same concentration to pericyte-depleted animals through the posterior semicircular canal at 1 week after pericyte depletion (illustrated in Figure 6, A and B). The distribution of AAV1-HRE-VEGFA165 vectors in the stria vascularis and expression of VEGFA in the cochlea were assessed from day 3 up to 2 weeks after viral delivery. We found the viral vectors were successfully transfected into the stria vascularis, as shown in Supplemental Figure 4. A significantly higher expression of *Vegfa* mRNA was found on day 3 and at 1 week in the AAV1-VEGFA165–injected cochleae than in the control group. Two weeks after gene delivery, we further assessed the VEGFA expression at the transcription and protein level and found a significantly higher expression of VEGFA at both mRNA and protein levels in the AAV1-VEGFA165–injected animals than in the control group, as shown in Figure 6, C and D. We next examined whether there was new vessel growth by determining changes in vascular density, pericyte population, and the number of new pericytes (5-ethynyl-2′-deoxyuridine–positive [EdU^+^] pericytes) in the control and AAV1-VEGFA165–treated groups. Our data revealed that upregulation of VEGFA165 strongly promotes pericyte survival and proliferation. The total pericyte population was significantly higher in the AAV1-VEGFA165–treated group relative to the control group (Figure 6E). A larger population of EdU^+^ pericytes was also found in the AAV1-VEGFA165–treated group than in the control group (Figure 6F and Supplemental Figure 5). In addition, significantly more EdU^+^ endothelial cells (ECs) and an EdU^+^ unknown cell type surrounding vessels were found in the AAV1-VEGFA165–treated group. Concurrent with the enhanced pericyte coverage and survival under AAV1-VEGFA165 gene transfection, vascular reduction in the AAV1-VEGFA165–treated group was significantly attenuated (Figure 6G). Figure 6, H and I, are representative confocal projection images showing blood vessels, EdU^+^ pericytes, ECs, and an unidentified cell type in the stria of the 2 groups. Figure 6J shows the different types of EdU^+^ cells highlighted in the zoomed-in images. Using IVM, and consistent with the improved morphometric data on the vessels, we found the AAV1-VEGFA165–treated group had less vascular leakage (Figure 6L) and significantly improved blood circulation compared with the control AAV1 group (Figure 6K). Figure 6, M and N, are representative images captured under IVM from the control and AAV1-VEGFA165–treated groups.

### Restoration of pericytes and vascular function significantly attenuates hearing loss.

**Figure 7:**
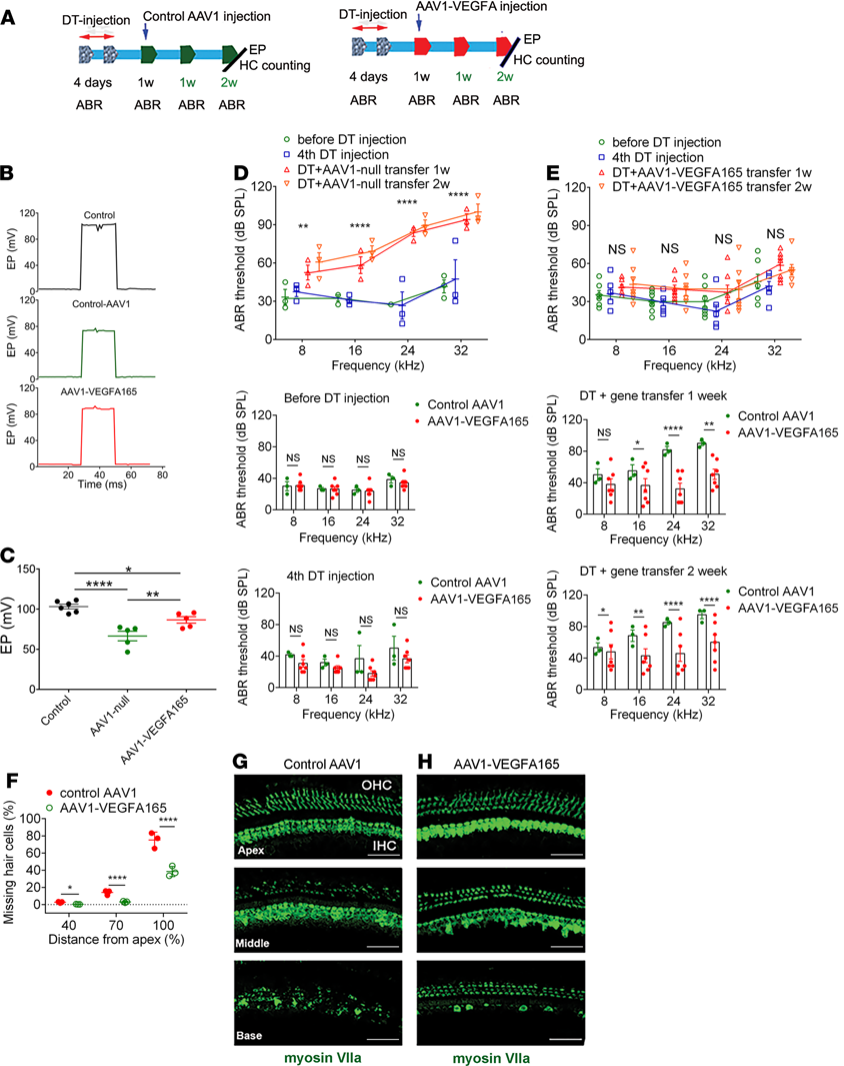
VEGFA165 gene therapy markedly attenuates the loss of HCs and improves hearing sensitivity. (**A**) An illustration of time points for gene delivery. (**B**) Representative EP waveforms from different groups. (**C**) Average EP in the AAV1-VEGFA165–transfected group is significantly higher than in the control and control AAV1 groups (*n* = 6 for the control, *n* = 5 for the AAV1 control, *n* = 5 for the AAV1-VEGFA165, *P < 0.05, **P < 0.01, and ****P < 0.0001 by 1-way ANOVA). (**D**) Hearing thresholds at different frequencies are significantly elevated after DT injection, but hearing sensitivity is not improved at 1 or 2 weeks in the control AAV1 gene group. In contrast, (**E**) hearing sensitivity is significantly improved at 1 week after AAV1-VEGFA165 gene delivery and further improved at 2 weeks (*n* = 3 for control group, *n* = 7 for AAV1-VEGFA165 group, **P < 0.01, and ****P < 0.0001 by Student’s *t* test). (**F**) Significant HC loss is seen at the middle and basal turns in the pericyte-depleted group, but HC loss is significantly attenuated in the AAV1-VEGFA165–transfected group (*n* = 3 for each group, *P < 0.05 for apical loss, and ****P < 0.0001 for middle and base HC loss by Student’s *t* test). (**G** and **H**) Representative confocal projection images of the organ of Corti in different cochlear regions recorded from a control AAV1 and AAV1-VEGFA165–treated group, demonstrating HC loss at the different cochlear turns. HCs were labeled with an antibody for myosin VIIa. Data are presented as mean ± SEM. EP, endocochlear potential; OHC, outer hair cell; IHC, inner hair cell. Scale bars: 50 μm.

Does the restoration of lost pericytes and vascular function attenuate hearing loss? In this study, we examined the EP, HC population, and hearing sensitivity 2 weeks after gene transfer. We found the pericyte regeneration and restoration of vascular function had significantly ameliorated EP, enhanced HC survival, and attenuated hearing loss. Figure 7A shows the time points of the gene delivery and related measurements. Figure 7, B and C, respectively show representative EP recordings in the control, pericyte-depleted + control AAV1–treated, and pericyte-depleted + AAV1-VEGFA165–treated animals and the average values of the EP in different groups. The EP did not improve in the control AAV1-treated group. However, the decline in EP was significantly ameliorated in the AAV1-VEGFA165 gene–treated group. Figure 7, D and E, show the AAV1-VEGFA165 treatment markedly improved hearing sensitivity at all measured sound frequencies 1 week after AAV1-VEGFA165 gene delivery, and hearing function further improved at 2 weeks after AAV1-VEGFA165 gene treatment compared with the control AAV1 group. In support of the improved hearing sensitivity, we found both OHC and IHC populations were higher in different cochlear turns of the AAV1-VEGFA165–treated group compared with the control AAV1 group. Figure 7F shows the plots of the total number of surviving HCs. Figure 7, G and H, are representative confocal images showing increased survival of HCs immunolabeled for the marker protein myosin VIIa in the control AAV1 and AAV1-VEGFA165–treated groups.

### VEGFA165 attenuates the reduction in strial vascular density and improves hearing sensitivity after acoustic trauma.

**Figure 8:**
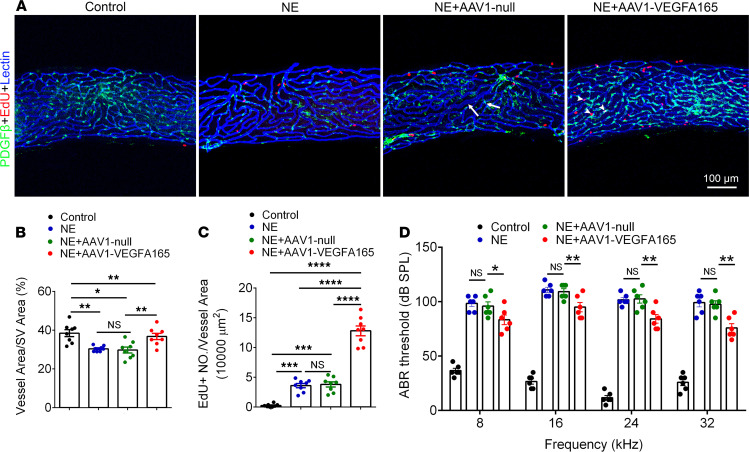
Therapeutic VEGFA165 gene treatment attenuates reduction in strial vascular density and improves hearing sensitivity after acoustic trauma. (**A**) Strial microvasculature labeled with lectin and EdU^+^ cells in control, 4 weeks after noise exposure (NE) without gene treatment, noise-exposed + control AAV1-null gene treatment, and noise-exposed + AAV1-VEGFA165 gene treatment 2 weeks after acoustic trauma groups. Arrows indicate vessel enlargement and shrinkage after noise exposure, and arrowheads show representative EdU^+^ pericytes, ECs, and an unidentified cell type in the stria. (**B** and **C**) Data analysis of EdU^+^ cells/vessel area and capillary density in control, NE, NE+AAV1 null (control); and NE+AAV1 V*EGFA165* (*n* = 8 for each group, *P < 0.05, **P < 0.01, ***P < 0.001, and ****P < 0.0001 by 1-way ANOVA). (**D**) Hearing thresholds at different frequencies are significantly elevated after noise exposure but improved after delivery of the AAV1-VEGFA165 (*n* = 6 for each group, *P < 0.05, and **P < 0.01 by 1-way ANOVA). Data are presented as mean ± SEM. Scale bars: 100 μm.

Destructive changes in strial capillaries (vessel shut down and intravascular strand formation) with exposure to loud sound have long been observed (23–27). Can VEGFA165 gene therapy reverse noise-induced vascular degeneration and improve hearing sensitivity? To test this, control AAV1 or AAV1-HRE-VEGFA165 was locally transplanted through the posterior semicircular canal to noise-exposed animals 2 weeks after acoustic trauma. We found that the transplantation of AAV1-VEGFA165 strongly promoted angiogenesis, as shown in Figure 8A. A larger population of EdU^+^ cells within or surrounding vessels was found in the AAV1-VEGFA165–treated mouse cochleae relative to the noise-exposed (without gene therapy) and noise-exposed (control AAV1-treated) groups (Figure 8B). The noise-induced capillary degenerative change was significantly attenuated in the AAV1-VEGFA165–treated group relative to the controls (Figure 8C). In parallel with the improved strial vasculature, the elevation of the hearing threshold was significantly ameliorated in the AAV1-VEGFA165 gene–treated group (Figure 8D).

## Discussion

In this study we demonstrated, for the first time to our knowledge, that cochlear pericytes are essential for vascular health and critical for hearing sensitivity in the adult cochlea. Pericyte loss caused vascular damage and blood-barrier breakdown with a corresponding loss of sensory HCs and hearing sensitivity. Using advanced in vitro tissue explant models, we found the pericytes signaled by VEGFA165 were indispensable for new vascular sprouting. VEGFA165 promoted pericyte-driven angiogenesis and enhanced pericyte survival under hypoxic conditions. In vivo, using AAV1-mediated VEGFA165 gene therapy, we provide compelling evidence that the upregulation of VEGFA165 improved vascular function by promoting pericyte survival and proliferation. The restoration of vascular function with gene therapy significantly attenuated the decline in EP, damage to sensory HCs, and loss of hearing sensitivity. Therapeutic targeting of pericytes with AAV1-mediated VEGFA165 gene therapy in an animal model provides a scientific foundation for future new clinical treatments. The new findings may also be relevant to the treatment of other diseases associated with the loss of pericytes (28–31).

### Pericyte loss in the adult cochlea leads to vascular dysfunction, loss of sensory HCs, and reduced hearing sensitivity.

Pericytes display heterogeneous morphological, biochemical, and physiological characteristics (32). However, the full range of organ-specific roles pericytes play is not well understood. In this study, we depleted pericytes in the cochlea in a genetically inducible/conditional adult murine model in which the *Pdgfrb-CreERT2*/iDTR mice express DTR under pericyte marker *Pdgfrb-Cre* control. The Cre-recombined PDGFR-β^+^ pericytes expressing DTR are selectively susceptible to the DT and ablated on exposure, while the Cre-negative murine cells, lacking DTR expression, are unaffected (33). We found the ablation of pericytes caused deleterious changes in the architecture of the cochlear vasculature, with visible enlargement and shrinkage in areas of pericyte loss or injury (as shown in Figure 1, H and I). The loss of pericytes also caused strial vascular leakage (Figure 1L). The vascular pathology was seen in different turns of the cochlea. Correspondingly, hearing loss was detected across the spectrum from low to high frequency, but the most severe loss was in high frequencies at 2 weeks after pericyte depletion. The depletion of pericytes also caused significant HC loss across all cochlear turns, but in particular it caused IHC loss in basal regions and regions toward the middle turn (Figure 2E). The cochlea is tonotopically organized, with high frequencies encoded at the base and lower frequencies encoded in apical locations (34). Increased sensory HC loss at the middle and basal turn indicates the particular vulnerability of these HCs to vascular dysfunction. Earlier studies from other laboratories have also shown HCs at the basal turn more susceptible to environmental perturbation (35, 36). The mechanisms underlying the loss of HCs were not investigated in this study. However, the pericyte loss–induced HC death could have resulted from the early reduction in blood flow associated with the hypoxia. Earlier, we noted that pericytes in the microvasculature of the spiral ligament work together with smooth muscle cells to control cochlear blood flow (37). However, we did not study pericytes in the region of the spiral ligament; they are a different phenotype and do not form part of the tight blood-barrier in the stria vascularis (3). Although we do assume the pericytes in the spiral ligament were also depleted (as those in the stria vascularis were), and would likewise have contributed to reduction in blood flow, our study focused on the differential effect of pericyte depletion in the strial blood–barrier. Vascular leakage, leukocyte infiltration, and later microvascular degeneration would also have contributed to HC death by further diminishing the capillary blood perfusion and accelerating the hypoxic inflammation in the cochlea. HCs are known to be extremely vulnerable to oxygen deficit (38), as well as sensitive to changes in ion concentration (39–41). In addition, a recent study has shown that injured pericytes release a variety of cytokines triggering tissue inflammation (42, 43). These factors could either singularly or synergistically contribute to HC death. In all cases, however, normal pericyte function and blood supply to the ear are critical for maintaining HC health and sustaining hearing acuity.

### Pericytes, signaled by VEGFA165, have strong vascular regenerative capacity in vitro.

**Figure 9:**
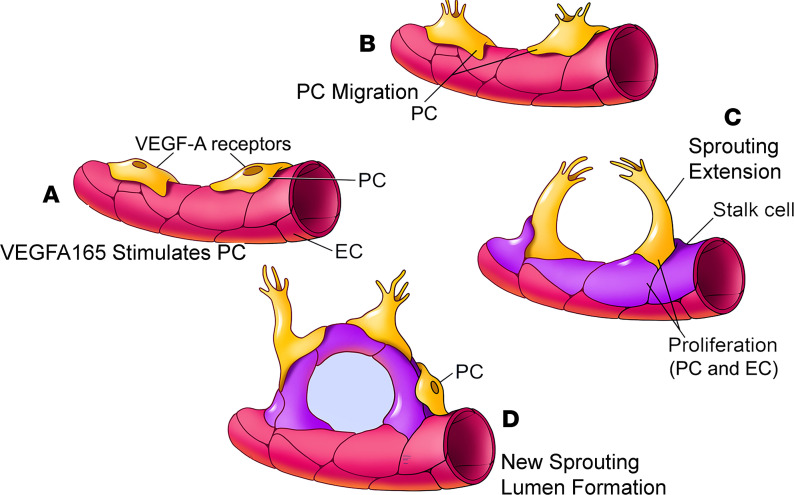
A model of VEGFA165-controlled angiogenesis in the cochlea. (**A** and **B**) VEGFA165 induces pericytes to migrate. (**C**) The pericytes “sense” the VEGFA165 diffusing from vessels and align along the VEGFA165 gradient to form a “sprout.” The proliferation of ECs behind the tip cells drives the precapillary to elongate. (**D**) Newly proliferated pericytes on the new branch tube establish focal contacts with ECs.

Pericytes are essential for vascular morphogenesis during normal development (18). Pericytes as precursors of mesenchymal stem/stromal cells have strong differentiation potential and can differentiate into a variety of cell types, including osteoblasts, adipocytes, and chondrocytes (44). However, it had not been known that pericytes are the leading cell in control of angiogenesis in the inner ear. An early study by Bergers and Song (45) demonstrated pericytes migrate ahead of endothelial cells and, by virtue of VEGF secretion, guide endothelial cell sprouting. In more recent work, Eilken et al. (46) showed pericytes regulate endothelial cell sprouting through VEGFR signaling. Teichert et al. (47) demonstrated that pericyte-expressed Tie2 controls angiogenesis and vessel maturation. Kang et al. (48) reported that pericytes enable effective angiogenesis in the presence of proinflammatory signals. It has long been known the first step in angiogenesis is the production of specialized tip cells. The tip cells have long and dynamic filopodia (“fingers that do the walking”) and display migratory behavior (18, 49, 50). Tip cells, as the leading cells in angiogenesis, guide endothelial cells and read environmental cues. Stalk cells (endothelial cells) trail behind the leading tip cells. In our observation, it was pericytes, not ECs, that became the tip cells and developed the filopodia to directly control new vessel growth in both adult and neonatal mouse cochleae, as shown in Figure 3. Ablation of pericytes significantly blocked the angiogenic activity. In this study, we also found the pericyte-led new vessel growth was strongly controlled by VEGFA165 signaling. The VEGFA165 mobilized cochlear pericytes to migrate from the tissue and guide endothelial cell extension and thus to initiate new endothelial branch formation. In contrast, the angiogenesis was significantly halted by blockage of the VEGFA165 signal (Figure 4). Based on our data, we hypothesize a new cochlear angiogenesis model, as illustrated in Figure 9. In the model, VEGFA165 induces pericytes to migrate, and the pericytes “sense” the VEGFA165 diffusing from vessels and align along the VEGFA165 concentration gradient to form a “sprout.” The proliferation of endothelial cells behind the pericytes drives the precapillary to elongate. Meanwhile, the proliferation of pericytes on the new branch tube establishes focal contacts with other endothelial cells so as to stabilize the new branch.

### Vegfa165 gene therapy enhances pericyte survival, promotes pericyte proliferation, attenuates vascular damage, and improves hearing sensitivity.

Can lost pericytes and vascular structure be restored by activation of VEGFA165 signaling in vivo? The VEGF family in mammals comprises 4 members: VEGFA, VEGFB, VEGFC, and VEGFD. VEGFA binding to its 2 receptors, Flt1 and Flk1, is more thoroughly studied than the others and is shown to be key for angiogenesis (10). In particular, VEGFA165, an endogenous COOH-terminal splice variant of VEGFA, is a specific angiogenic peptide (51), which plays a critical role in stimulating angiogenesis (10). VEGFA165 delivered at an appropriate microenvironmental concentration has been shown to act as a regulator in vascular proliferation and new vessel formation (22). In this study, we demonstrate that a single injection of an AAV1-VEGFA165 viral vector into the perilymphatic system promoted pericyte survival, increased both pericyte and endothelial cell proliferation, and prevented the loss of vascular volume. In addition to the role of VEGFA165 as a promoter of angiogenesis, we also found the VEGFA165 to enhance pericyte survival under hypoxic conditions in vitro, as shown in Figure 5. The accumulation of EdU^+^ pericytes was found in damaged vascular areas of therapeutically transfected AAV1-HRE-VEGFA165 animals. We also noticed that the VEGFA165 promoted the proliferation of ECs and an unidentified perivascular cell. The perivascular cell is particularly interesting as it is not a pericyte, neither expressing the pericyte marker protein, PDGFR-β, nor situated on the vessel wall (the vessel wall is traditionally regarded as only having a structural role; however, recent studies indicate the vessel wall contributes to repair and regeneration as a stem cell reservoir) (52, 53). Identification of the new cellular phenotype will be potentially important for strial regenerative medicine in the future. Maintenance of normal blood flow and vascular integrity is critical for cochlear ion homeostasis, in particular for maintaining the EP, which is critically important for normal hearing function (13, 54). It is expected that restoration of failed vascular function attenuates loss in EP, promotes HC survival, and improves hearing sensitivity, as shown in Figure 7. Here we should also point out that increased sensory HC survival (particularly IHC survival) and improved hearing function could be a function of pericyte survival and regeneration, as pericytes are known to release a number of growth factors, including fibroblast growth factor, brain-derived neurotrophic factor, and pericyte-derived pleiotrophin (17, 55). These growth factors substantially affect overall organ health, particularly neuron health, as recently shown by Nikolakopoulou et al. (17). The direct adverse impacts of pericyte loss on auditory neuron survival, as opposed to the downstream effect of pericyte loss on the vasculature, were not investigated in this study. A future study using a coculture of pericytes with sensory HCs would verify the direct effect of pericytes on sensory auditory neuron health and pathology. Nevertheless, the existence of any additional collateral effects does not detract from our central conclusion supporting the vital role that pericytes play in the formation and maintenance of lateral wall vascular networks and hearing.

Consistent with our previous report (56), AAV1-VEGFA165 gene therapy also attenuated reduction of strial microvasculature and improved hearing sensitivity (Figure 8). The level of loud sound used in the study would be expected to cause both mechanical and metabolic (hypoxic) damage to vascular and nonvascular cells (HCs) in the cochlea. While the restoration of vascular function would not be expected to restore lost sensory HCs, it could, with rapid reestablishment of blood supply, prevent further HC death, stabilize residual hearing after damage, and potentially accelerate hearing recovery. Transfection with AAV1-HRE-VEGFA165 was shown to markedly promote vascular volume and blood flow, increase proliferation of pericytes and ECs, and attenuate loud sound–caused loss in EP and hearing. Our results indicate that loud sound–triggered transformation of pericytes contributed to capillary wall thickening and regression, while AAV1-VEGFA165 gene therapy effectively rehabilitated the vascular regression and improves hearing.

In conclusion, the data provide compelling evidence that pericyte health is critical for blood-tissue barrier stability/integrity and hearing in the adult ear. Pericytes, modulated by VEGFA165 signaling, control cochlear health and angiogenesis. Restoration of pericytes improves vascular function, prevents loss of sensory HCs, and attenuates the deficit in hearing. AAV1-mediated VEGFA165 gene therapy in the inner ear has strong potential for treating pericyte deficiency such as from acoustic trauma. The new findings may also be relevant to preventing or restoring neuron loss in other diseases such as neurological disorders in the brain and retina.

## Methods

### Animals.

All strains of mice used in this study were originally purchased from The Jackson Laboratory (details in the Supplemental Methods). Briefly, conditional pericyte depletion *Pdgfrb-CreERT2*/iDTR mice were created by crossing a Cre-driver line: B6.Cg-Tg (*Pdgfrb-cre/ERT2*)6096Rha with C57BL/6-Gt (57) 26Sor^tm1(HBEGF)Awai^ carrying an inducible human diphtheria toxin receptor allele (iDTR) preceded by a *loxP*-flanked stop cassette. To verify location of the PDGFR-β–Cre, tdTomato fluorescence reporter mice were created by breeding B6.Cg-Gt (57) 26Sor^tm9(CAG-tdTomato)Hze^ with B6.Cg-Tg (*Pdgfrb-cre/ERT2*)6096Rha. Cre-mediated recombination was initiated by intraperitoneal injection of tamoxifen at 75 mg/body weight at age P32, every 24 hours for 3 consecutive days. To deplete pericytes, *Pdgfrb-CreERT2*/iDTR and its control (iDTR) mice were given DT intraperitoneally once every 24 hours at a dose of 10 ng/g on 4 consecutive days after tamoxifen administration. All transgenic mice in this study were validated and genotyped. Both male and female mice were used, and all mice used were adults aged 4–8 weeks or P1–P15.

### Noise exposure and auditory brainstem response.

Animals (at ages 6–8 weeks, both sexes) were placed in wire mesh cages and exposed to broadband noise at 120 dB sound pressure level in a sound exposure booth for 3 hours and for an additional 3 hours the following day. The routinely used noise exposure regimen produces permanent loss of cochlear sensitivity. ABR audiometry to pure tones was used to evaluate hearing function in control, noise exposure, and noise exposure + gene delivered groups as previous reported (56) (details in the Supplemental Methods).

### Assessment of pericytes, vascular density, and vascular permeability.

Mice were anesthetized and placed on a water heating pad. Fluorescent dye Lectin–DyLight 649 (DL-1178, Vector Laboratories) diluted in 0.1 M PBS buffer to a concentration of 20 μg/mL (vol. 100 μL) was administered via intravenous retro-orbital sinus to the animals 5 minutes before they were sacrificed. Cochleae were harvested and fixed in 4% paraformaldehyde (58) overnight. Whole mounts of stria vascularis were carefully isolated and permeabilized in 0.5% Triton X-100 (MilliporeSigma) for 30 minutes and blocked with 10% goat serum (G9023, MilliporeSigma) for 1 hour. The specimens were then incubated with a monoclonal primary antibody, rabbit anti–PDGFR-β [Y92] (ab32570, 1:50, Abcam), in 1% BSA in PBS overnight at 4°C. After 3 washes in PBS, samples were incubated with the secondary antibody, Alexa Fluor 488–conjugated goat anti–rabbit IgG (A11008, Invitrogen, Thermo Fisher Scientific), for 1 hour at room temperature. After three 30-minute washes in PBS, the tissue was mounted in medium (H-1000, Vector Laboratories) and visualized under an FV1000 Olympus confocal microscope with a 40× objective (Olympus FV1000). For each cochlea, images were recorded at 3 randomly chosen, nonoverlapping locations for each turn along the approximately 300 μm length of the stria vascularis. The pericyte number and blood vessel area were calculated using Fiji (ImageJ, NIH, 1.51t) software. The pericyte population was defined as pericyte density = number of pericytes/area of blood vessel. Vascular density was analyzed as previously described (14) and was defined as vascular density = area of blood vessels/area of stria vascularis. Briefly, the channel uniquely showing vessels was separated from the confocal image, and the threshold adjusted to maximize vessel intensity relative to the background. The pixel area of the stria vascularis was determined under a differential interference contrast (DIC) microscope. The vascular density was assessed as a percentage of vascular pixel area/pixel area of stria vascularis in each measured area and at each turn. The data were averaged as mean ± SEM determined from 6 (control) or 7 (pericyte-depleted) whole-mounted specimens. For noise exposure, the vascular densities were determined from whole-mounted specimens of the control, noise-exposed, noise-exposed + control AAV1–treated and noise-exposed + AAV1-VEGFA165–treated animals (*n* = 8). Vascular permeability in the control and pericyte-depleted animals was assessed using an FITC-conjugated bovine albumin tracer (FITC-albumin, 66 kDa, A-9771, MilliporeSigma). Specifically, vascular permeability in vivo was assessed by recording tracer movement observed through the prepared open vessel window, as previously reported (59).

### Quantification of capillary diameter.

The diameter of the capillaries was determined from images acquired with an FV1000 Olympus laser-scanning confocal microscope. ImageJ software was used to determine the diameter of the capillary. In analyzing the pericyte effect on the blood vessel structure, the measurement of both the minimum and maximum capillary diameters were recorded. The diameter value was calculated by averaging 3 vessels in each image. The data were averaged as mean ± SEM determined from 6 (control) or 7 (pericyte-depleted) whole-mounted specimens (a total of 51 images for the control group, 60 images for the pericyte-depleted group).

### Ex vivo strial tissue explant and angiogenesis.

The stria vascularis was isolated from the cochlea, trimmed and cut into small pieces approximately 2–3 mm^3^ in size, embedded in a 3D Matrigel matrix–precoated dish, and bathed in culture media containing VEGFA165 (50 ng/mL) for 4 or 5 days. For the cell-tissue coculture experiment, pericytes encoded with fluorescence protein pmOrange2 at 1.0 × 10^5^ cells/mL were transplanted to the strial explant on day 4 in culture medium for 24 hours. For depletion of pericytes, tissue explants were pretreated with APB5 at a concentration of 5 μg/mL, 8 μg/mL, and 10 μg/mL 1 day prior to VEGFA165 treatment and maintained in the medium until the end of the experiment. The culture medium was changed every 2 days to ensure constant agent concentration. For the tissue explant from neonatal mice, new vessel growth was recorded every day for 4 or 5 days under DIC and confocal fluorescence microscopy. To assess proliferation, EdU (10 μM, C10638, Invitrogen, Thermo Fisher Scientific) was added to the culture medium on the first day of the experiment. New vessel structure was determined from fluorescence F-actin labeled with phalloidin conjugated to different fluorophores Alexa Fluor (A12379, 488; A12380, 568; A22287, 647; Life Technologies, Thermo Fisher Scientific). The branch number and length of new vessels per unit area were quantitatively assessed using ImageJ. For quantification of branch number, we scored total sprouts per sample using the method of Hellström (11) over the whole area of the explant (new branch formations outside the tissue explant). For the 3D rendering of the confocal data, we employed Fiji and UCSF Chimera (http://www.cgl.ucsf.edu/chimera/) for visualization, processing, analysis, and presentation. Individual channels were split to separate image stacks and filtered to reduce the signal-to-noise ratio. Volume data were displayed at respective thresholds as solid or partially transparent isosurfaces in UCSF Chimera using its volume viewer.

### Viral vector constructs.

Two viral vectors, Cre-dependent pAAV-CAG-flex-rev-hVEGFA165-P2A-GFP and pAAV-5HRE-CAG-VEGFA165-P2A-GFP, were designed in the lab and constructed at Oregon Health and Science University (OHSU). The pAAV-CAG-flex-rev-hVEGFA165-P2A-GFP was constructed by replacing rev-oChIEF-tdTomato in pAAV-Flex-rev-oChIEF-tdTomato (Addgene plasmid 30541) with rev-hVEGFA165-P2A-GFP, and synthesized at GenScript. Because the sequence coding for hVEGFA165-P2A-GFP was reversed and floxed by 2 incompatible *LoxP* sites, hVEGF-A_165_-P2A-GFP is only expressed in infected cells also expressing Cre recombinase. The pAAV-5HRE-CAG-VEGFA165-P2A-GFP was constructed by replacing EF1a-DIO-oChIEF (E163A/T199C)-P2A-dTomato in pAAV-EF1a-DIO-oChIEF (E163A/T199C)-P2A-dTomato-WPRE-BGHpA (Addgene plasmid 51094) with 5HRE-CAG-VEGFA165-P2A-GFP and synthesized at GenScript. All recombinant AAV serotype 1 vectors were produced at OHSU. Titer of rAAV-CAG-flex-rev-hVEGFA165-P2A-GFP was 1.4 × 10^13^ genome copies/mL and pAAV-5HRE-CAG-VEGFA165-P2A-GFP 5.1 × 10^13^ viral genomes/mL (vg/mL).

### VEGFA165 blocking and genetic manipulation of VEGFA165 production by pericytes.

To block the VEGFA165 signal, tissue explants were pretreated with BAW2881 (S8189, Selleckchem), a VEGF receptor tyrosine kinase inhibitor, at 1 nM, 4 nM, and 40 nM, prior to VEGFA165 treatment and maintained in the medium for 5 consecutive days. On day 5, tissue explants were fixed in 4% PFA for 3 hours at room temperature, rinsed in PBS, permeabilized in 0.5% Triton X-100 for 1 hour, immunoblocked with a solution of 10% goat serum in 1% BSA-PBS for 1 hour, and then incubated with Alexa Fluor 568 phalloidin (A12380, Life Technologies, Thermo Fisher Scientific) at 1:200 for 2 hours. After 3 washes in PBS, the tissues were visualized under a confocal microscope, and the number of branches was assessed (3–4 samples/group). For the quantification of branches, the tissue area was determined using ImageJ, as described above. For genetic manipulation of VEGFA165 production in the pericytes, *Pdgfrb-Cre*/tdTomato fluorescence reporter mice were used. Cre-mediated recombination was initiated by an intraperitoneal injection of tamoxifen at 75 mg/body weight at age P32, every 24 hours for 3 consecutive days. The stria vascularis was isolated from the cochlea and embedded in a 3D Matrigel matrix to make an angiogenesis model. A total of 2 μL of Cre-dependent pAAV1-CAG-flex-rev-hVEGFA165-P2A-GFP vector (1.4 × 10^13^ vg/mL) or its control vector was added to the culture medium the next day for 4 consecutive days. Tissue from control and AAV1-VEGFA165–transfected groups was fixed on day 5 and double immunolabeled with GFP-Booster Atto488 (gba488-100, Chromotek) and phalloidin for visualizing the branch structure.

### AAV1 viral vector transfection of pericytes in vitro.

The primary pericyte cell line was generated from C57BL/6J mouse cochleae by a well-established minichip protocol previously described and published (60). Pericytes at passage 3 were grown in 24-well plates in 500 μL of culture medium. The next day, the cells were transfected with either AAV1-GFP (control AAV1-null) or AAV1-HER-VEGFA165 at 1 × 10^4^, 5 × 10^4^, 1 × 10^5^, and 3 × 10^5^ multiplicity of infection (MOI) for 48 hours. The cells were observed and recorded under an Olympus Fluoview FV1000 confocal microscope. The density of AAV1-GFP expressing cells was determined to evaluate the transfection efficiency. To determine the effect of AAV1-HER-VEGFA165 on pericytes under hypoxia, pericytes were divided into 4 groups, including control, pericytes transfected with AAV1-GFP, pericytes transfected with AAV1-HER-VEGFA165 under normoxia, and pericytes transfected with AAV1-HER-VEGFA165 under hypoxia. Each group of 1 × 10^5^ purified pericytes was plated in a 35 mm dish and transfected with AAV1-HER-VEGFA165 (AAV1-GFP as control) at an MOI of 1 × 10^5^ for 48 hours. After changing the medium, the hypoxia group was placed in a hypoxic incubator filled with a gas mixture of 5% O_2_ + 10% CO_2_+ 85% N_2_) for 1 hour at 37°C. At the end of the study, cells from different groups were lysed, and RNA from each sample was extracted using RNeasy Micro Kit (74004, Qiagen) per the manufacturer’s recommendation. The sample was reverse-transcribed with a RETROscript kit (Ambion), and cDNA synthesized from total RNA was diluted 10-fold with DNase-free water; each cDNA sample was independently measured 3 times. Transcripts were quantified by TaqMan Gene Expression Assay for *Vegfa* (Mm01281449_m1) on a model 7300 real-time pericyte system (Applied Biosystems, Thermo Fisher Scientific). The real-time PCR was cycled at 95°C for 20 seconds, 40 cycles of 95°C for 1 second, and 60°C for 20 seconds. Mouse *Gapdh* served as the endogenous control. Quantitative pericyte analysis was performed per the guidelines provided by Applied Biosystems, Thermo Fisher Scientific, using a comparative cycle threshold method.

### Delivery of the AAV1 viral vector to the cochlea in vivo.

Mice 1 week after the final DT (or mice 2 weeks after noise exposure) were anesthetized and maintained at 37°C with a circulation water heating pad (Kent Scientific). Animal eyes were protected with a lubricating ointment. Surgery was performed under an operating microscope. Briefly, a postauricular skin incision was made to access the temporal bone by making a small hole with a 30-gauge syringe needle in both posterior semicircular canal and lateral semicircular canal and leaving the hole open for a couple of minutes until no perilymph leakage was obvious. A total of 2 μL of AAV1-HRE-VEGFA165 vector suspension (5.1 × 10^13^ vg/mL) or control vector was injected into the posterior semicircular canal at a rate of 240 nL/min using a micropipette electrode connected to a standard syringe (MINJ-PD-S100, Tritech Research) and controlled by a microINJECTOR All-Digital Positive Displacement System (MINJ-PD) (58, 61, 62). The viral distribution in the stria vascularis was examined at day 3, 1 week, and 2 weeks after gene delivery in confocal images of cryostat sections of the whole-mounted cochleae. The details of cryostat sectioning were reported by Jiang et al. (63).

### Assessment of VEGFA expression and vascular cell proliferation in vivo.

Total RNA (300~500 ng) from the stria vascularis of AAV1-GFP control and AVV1-HRE-VEGFA165 groups was extracted using an RNeasy Mini Kit (74104, Qiagen) per the manufacturer’s recommendation. mRNA expression of *Vegfa* was measured by quantitative reverse transcription PCR as described above. To determine the expression of VEGFA at the protein level, the VEGFA concentration in the stria vascularis from different groups was measured using a VEGFA ELISA kit (ab119565, Abcam) at 2 weeks after transfection. Stria were isolated and homogenized in cell extraction buffer with a protease inhibitor cocktail on ice for 20 minutes and centrifuged at 8000*g* for 15 minutes at 4°C, and the supernatant was collected. To identify new blood vessels, EdU (A10044, Invitrogen, Thermo Fisher Scientific) was intraperitoneally injected to animals at 25 mg/kg body weight 1 day after gene delivery and subsequently every 2 days until sacrifice. To label ECs, mice were anesthetized and received 100 μL Lectin-DyLight 649 at 20 μg/mL by i.v. before they were sacrificed. The whole mounts of stria vascularis were isolated for immunohistochemistry as described above. For identification of pericytes, the specimens were incubated with antibody for PDGFR-β (Y92) (ab32570, 1:50, Abcam). The tissue was examined under a confocal microscope. The proliferating cells in the stria were assayed with a Click-iT Plus EdU Alexa Fluor 555 imaging kit (C10638, Life Technologies, Thermo Fisher Scientific) used per the manufacturer’s recommendation. The population of EdU^+^ cells was assessed at each cochlear turn along the approximately 300 μm length of the stria vascularis. The EdU^+^ cell density was defined as EdU^+^ cell density = number of EdU^+^ cells/area of stria.

### HC count.

Animals from DT-treated + AAV1-GFP and DT + AAV1-HRE-VEGFA165 groups were sacrificed, with cochleae quickly removed and then decalcified (1212, StatLab) overnight on a rocker at 4°C. Each decalcified cochlea was microdissected into 3 pieces. For HC counts, the tissues were permeabilized in 0.5% Triton X-100 (MilliporeSigma) for 30 minutes, blocked in 10% normal goat serum and 1% BSA-PBS for 1 hour, and stained with a rabbit anti–myosin VIIa primary antibody (25-6790, 1:200, Proteus) at 4°C overnight and the secondary antibody, goat anti–rabbit antibody, conjugated to Alexa Fluor 488 (ab150077, Abcam), for 1 hour. Samples were mounted on glass coverslips with mounting medium (H-1200, Vector Laboratories) and visualized under a confocal microscope. Low-magnification images of the whole mounts were acquired and localization of frequency determined based on the percentage distance from the base using the ImageJ Plugin, Measure_Line class (64–67). The total number of HCs was counted along the entire length of the basilar membrane from the apex to the base. Cytocochleograms were constructed by plotting the percentage of total HCs, and IHC and OHC loss, as a function of the percentage distance from the apex of the cochlea.

### EP measurement.

EP was measured in the control, DT + control AAV1, and DT + AAV1-HRE-VEGFA165 groups as previously described and reported (68) with a minor modification. Briefly, a silver chloride reference electrode was placed under the skin and the bulla exposed and perforated, exposing the basal turn of the cochlea. A glass micropipette filled with 150 mM KCl was advanced to the round window membrane and the offset adjusted to 0 baseline. Entry of the electrode tip into the scala media is characterized by the positive potential jump during recording. The pipette was advanced until a stable potential was observed. The potential was amplified (model 3000 AC/DC differential amplifier, A-M Systems), recorded via an A-D converter (Fluke II multimeter), and computer-recorded. The collected data were exported, waveform-reconstructed, and analyzed in SignalExpress 2015 (NI).

### Intravital fluorescence microscopy.

The mice from the DT + AAV1-GFP and DT + AAV1-HRE-VEGFA165 groups (2 weeks after gene delivery) were anesthetized, wrapped in a heating pad, and maintained at a rectal temperature of 37°C. A vessel-window was created using a small knife blade to scrape the lateral wall bone until a thin spot was cracked (for details, see Shi et al., ref. 59). The bone chips were removed with small wire hooks. The vessel-window was covered with a cut coverslip (12-542A, Thermo Fisher Scientific) to preserve normal physiological conditions and provide an optical view for recording vessel images. Vessels were visualized using FITC-dextran (2000 kDa, MilliporeSigma, FD2000s) as a contrast medium in the bloodstream. FITC-dextran was administrated intravenously to the mice at a concentration of 40 mg/mL in 0.1 mL physiological solution over a 5-minute interval. Strial images were recorded with a Zeiss LSM 7MP system with a long working distance objective (8.4 mm, 10×) at 2 frames. Blood flow was directly observed in real time on the video monitor. More than 350 images were acquired per video to ensure successful analysis of flow velocity. Vascular diameter and blood flow velocity, determined off-line from the captured video frames, were analyzed by a cross-correlation method using ImageJ. In brief, the luminal intensity of spatial structure in the image sequences was cross-correlated and blood flow velocity calculated by tracing moving FITC-labeled particles in the spatial distance between image locations by the time difference [velocity = distance(μm)/time(s)] (69). Volume flow (F) was estimated with the equation F = V × A (V, velocity; A, cross-sectional area of the vessel of πr^2^, where r = diameter/2). Vascular velocity and vascular volume were averaged as means ± SEM.

### Statistics.

All data are presented as mean ± SEM. SPSS 22.0 (or 25.0) was used for statistical analysis, and GraphPad Prism 8.0 software was used for the graphing. All data were tested for normality using a Shapiro-Wilk test. Statistical analysis was performed using 2-tailed Student’s unpaired *t* test under a normal distribution. One-way ANOVA was used for comparison among 3 or more groups. The repeated-measure ANOVA was used for the analysis of the changes in hearing loss between the groups. *P* less than 0.05 was considered significant: *P < 0.05, **P < 0.01, ***P < 0.001, and ****P < 0.0001.

### Study approval.

All animal experiments reported were approved by the OHSU Institutional Animal Care and Use Committee (IACUC TR01_IP00000968).

## Author contributions

JZ, ZH, XW, and HJ were involved in all aspects of the experiments and contributed equally. This appraisal is based on the number of experiments each of these members conducted, the unique experimental skills each brought to the experiments, the quality of their work, and their participation in the experimental design and discussion of the data. JZ, ZH, HJ, and LN were involved in the immunohistochemistry, reverse transcription PCR and ELISA, animal breeding, genotyping, and ABR test. YZ, ZH, and QY were also involved in noise exposure, animal breeding, genotyping, and cell counting. In addition, ZH performed the IVM. JZ, XW, and ZH performed the in vitro study and were involved in the data analysis. JZ and ZH formatted the references. JS performed the 3D reconstruction. MA provided critical insights into 3D data reconstruction. AF, GB, and MH provided valuable insights for data interpretation and discussion. XS supervised the experiments and wrote the manuscript.

## Supplementary Material

Supplemental data

## Version 1 03/09/2021

In-Press Preview

## Version 2 04/22/2021

Electronic publication
