# Supplementary material for: VEGFA165 gene therapy ameliorates blood-labyrinth barrier breakdown and hearing loss
Source: JCI Insight. 2021 Apr 22;6(8):e143285. doi: 10.1172/jci.insight.143285 (PMC8119217; doi:10.1172/jci.insight.143285)
Supplement: Supplemental data [file jciinsight-6-143285-s054.pdf]

Supplemental material

***Animals*.** All strains of mice used in this study were purchased from Jackson Laboratory, including the C57BL/6J (stock# 000664), Tg(Cspg4-DsRed.T1)1Akik/J (stock# 008241), B6;FVB-Ifi208$^{Tg(Cspg4\text{-}cre)1Akik}$/J (stock# 008533), B6.Cg-Gt(ROSA)26Sor$^{tm6(CAG\text{-}ZsGreen1)Hze}$/J (stock# 007906), B6.Cg-Gt (ROSA)26Sor$^{tm9(CAG\text{-}tdTomato)Hze}$/J (stock# 007909), B6.Cg-Tg (Pdgfrb-cre/ERT2)6096Rha/J (stock# 029684), and C57BL/6-Gt (ROSA) 26Sor$^{tm1(HBEGF)Awai}$/J(Stock# 007900). All transgenic mice were inbred in the lab. The neural/glial antigen 2 (NG2)-derived fluorescence reporter (green) NG2$^{+ZsGreen}$ transgenic mouse line was produced by crossing B6; FVB-Ifi208$^{Tg(Cspg4\text{-}cre)1Akik}$ with B6.Cg-Gt(ROSA)26Sor$^{tm6(CAG\text{-}ZsGreen1)Hze}$, NG2-derived pericyte fluorescence reporter (red) NG2$^{+DsRedBAC}$ mice by crossing C57BL/6 with Tg (Cspg4-DsRed.T1)1Akik, double pericyte fluorescence reporter (red and green) NG2$^{+DsRedBAC/ZsGreen}$ mice by crossing NG2$^{+DsRedBAC}$ with NG2$^{+ZsGreen}$, double fluorescence reporter NG2$^{+DsRedBAC/GFP}$ mice by crossing NG2$^{+DsRedBAC}$ with Tg(TIE2GFP)287Sato (003658).

***Auditory Brainstem Response (ABR)*.** ABR audiometry to pure tones was used to evaluate hearing function before and after DT treatment. ABR audiometry to pure tones was also used to evaluate hearing sensitivity in control and noise-exposed groups. Specifically, each animal was anesthetized with ketamine hydrochloride (100 mg/kg), and 2% xylazine hydrochloride (10 mg/kg, Abbott Laboratories), and placed on a heating pad (37°C) in a sound-isolated chamber. The external ear canal and tympanic membrane were inspected using an operating microscope to ensure the ear canal was clean and free of wax and deformity, the tympanic membrane clear of inflammation, and there was no effusion in the middle ear. Needle electrodes were placed subcutaneously near the test ear, at the vertex, and on the contralateral ear. Each ear was stimulated separately with a closed tube sound delivery system sealed into the ear canal. The auditory brain-stem response to a 1-ms rise-time tone burst at 8, 16, 24, and 32 kHz were recorded, and thresholds were obtained for each ear.

Supplemental results

Results

Figure 1

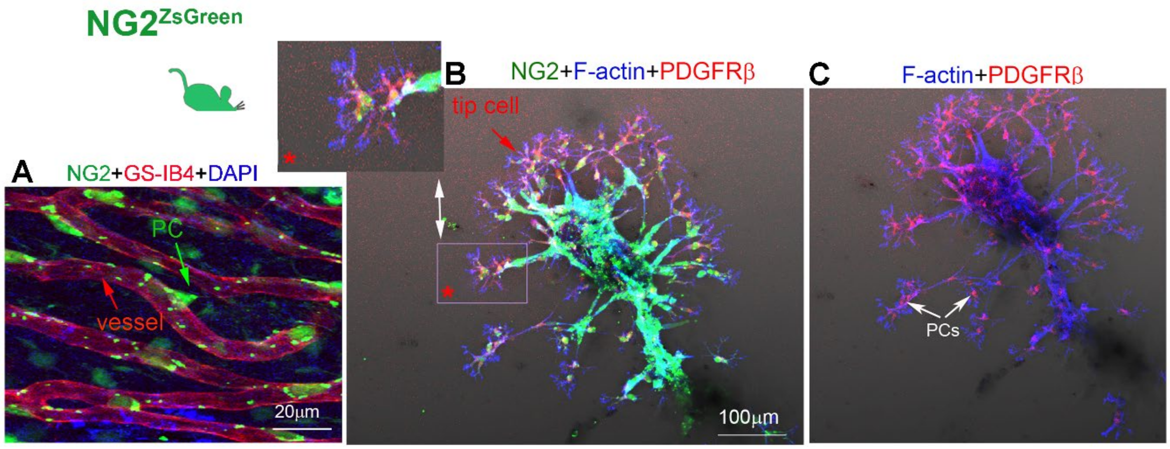

Figure 1. NG2-derived pericyte control of angiogenesis in the adult cochlea of a *NG2$^{+ZsGree}$* fluorescence reporter mouse. (**A**) A confocal projection image of the stria vascularis shows NG2-derived PCs (green) located on microvessels labeled with GSIB4 (red). (**B**) A confocal projection image of the strial explant of an NG2$^{+ZsGreen}$ mouse cochlea on day 5 after treatment with VEGFA165, triple labeled with Alexa Fluor® 647 Phalloidin, DAPI, and antibody for PDGFRβ. A high magnification image (*) in panel c highlights tip cells are NG2 (green) and PDGFRβ positive. (**C**) A single channel of the strial explant from image panel B further demonstrates the tip cell is positive for the PC marker protein, PDGFRβ.

Figure 2

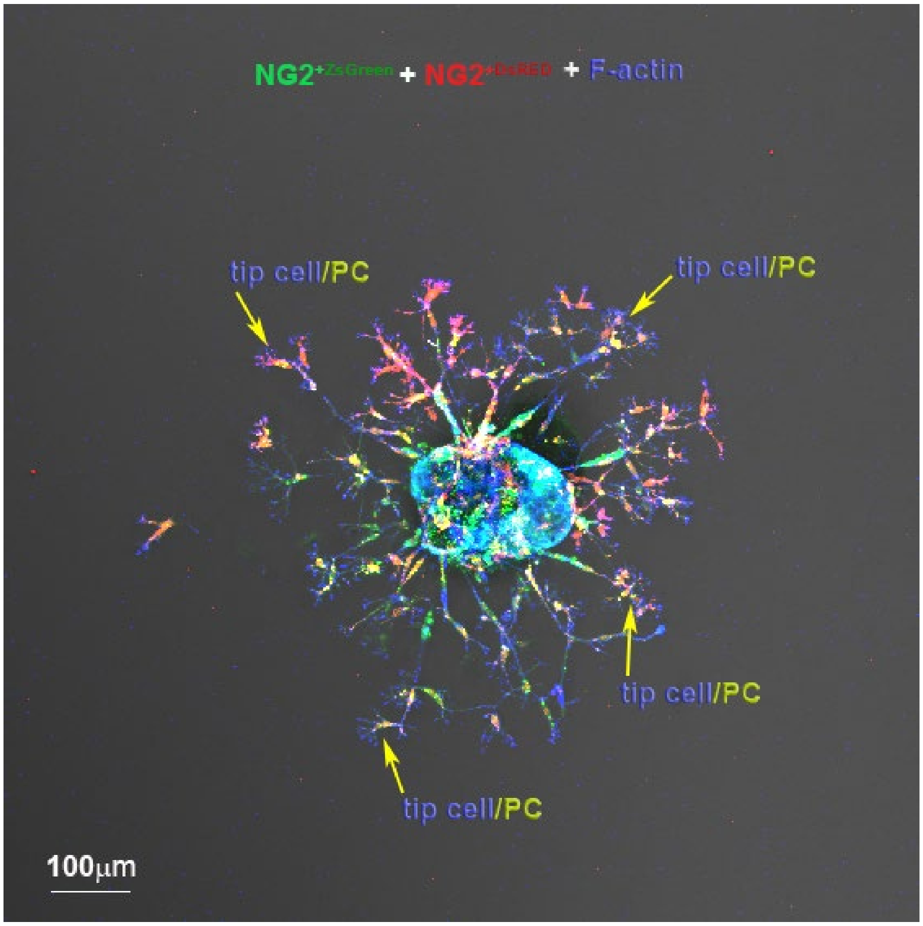

Figure 2. NG2-derived pericyte control of angiogenesis in the adult cochlea in a *NG2*$^{+DsRedBAC/ZsGreen}$ double fluorescence reporter mice. Confocal fluorescence combined with DIC image shows that strial explant from a double-fluorescent reporter *NG2*$^{+DsRedBAC/ZsGreen}$ mouse cochlea labeled with Alexa Fluor® 647 Phalloidin (blue) on day 5 after VEGFA165 treatment. We noticed that tip cells (yellow) are both NG2 (green) and NG2 (red) (Note: *NG2*$^{+DsRedBAC/ZsGreen}$ mouse was created by crossing the *NG2*$^{+DsRedBAC}$ with *NG2*$^{+ZsGreen}$).

Figure 3

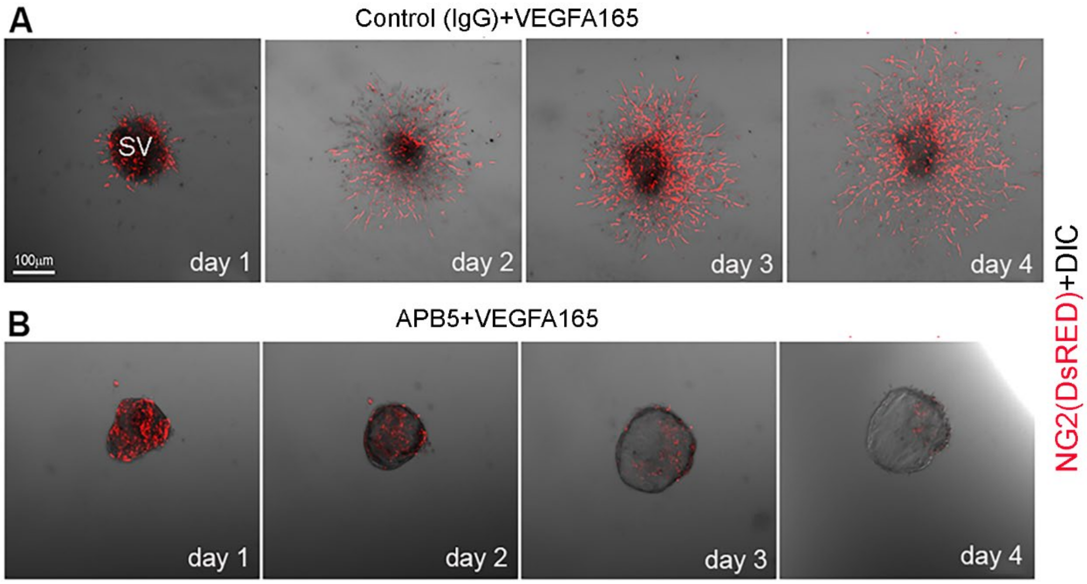

Figure 3. Sprouting angiogenesis in a native strial explant from P1 neonatal mouse cochleae. (**A**) and (**B**) Representative time-course imaging of sprouting angiogenesis in control and pericyte-depleted groups (SV: stria vascularis).

Figure 4

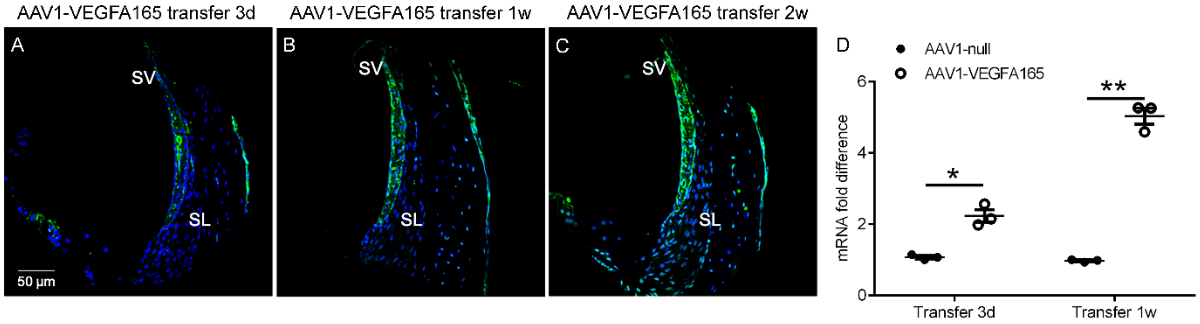

Figure 4. Expression of the AAV1-based VEGF-A_{165} vector in the stria vascularis was examined from day three up to two weeks after viral delivery. (**A-C**) show the AAV1 viral vector successfully transfected in the stria vascularis. (**D**) shows Significantly higher expression of VEGFA165 mRNA was found on day three and one week in the AAV1-VEGFA165 injected cochleae than in the control AAV1 group.

Figure 5

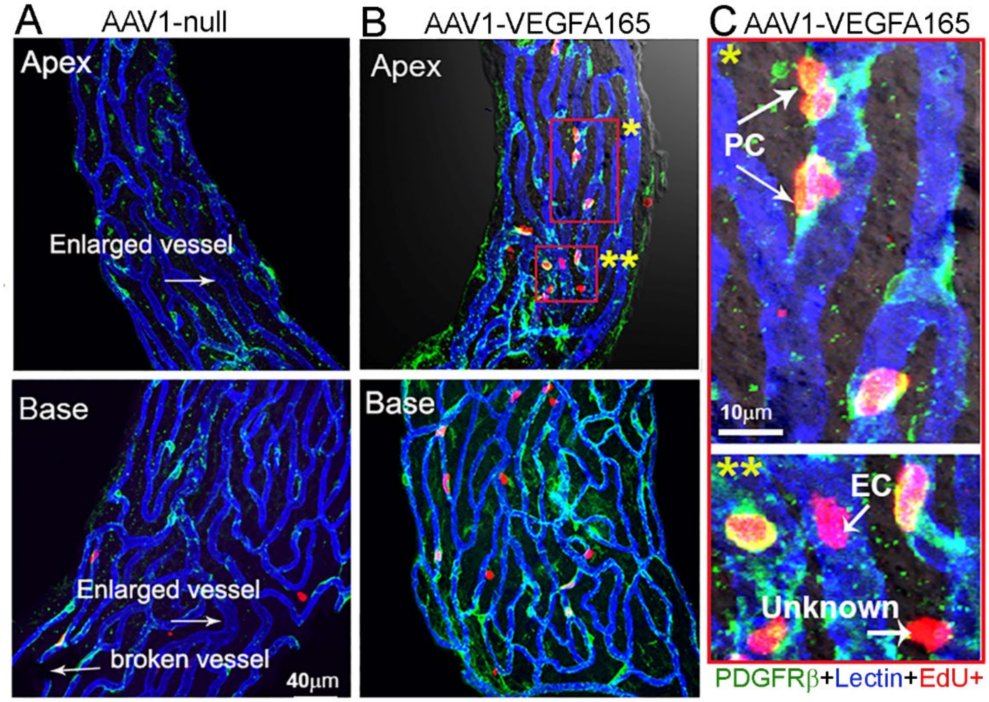

Figure 5. VEGFA165 gene therapy enhances pericyte survival and attenuates vascular damage. (**A**) and (**B**) Confocal projection images show EdU$^+$ pericytes, endothelial cells, and an unidentified cell type in the stria vascularis of control AAV1 (**A**, upper/lower) and AAV1-VEGF165 groups (**B**, upper/lower). (**C**) Zoomed-in images of EdU$^+$ positive cells from inserts (* or **) in (**B**). PC: perciyte, EC: endothelial cell.
